# Anti–PD-1 and Anti–PD-L1 in Head and Neck Cancer: A Network Meta-Analysis

**DOI:** 10.3389/fimmu.2021.705096

**Published:** 2021-08-09

**Authors:** Andrea Botticelli, Alessio Cirillo, Lidia Strigari, Filippo Valentini, Bruna Cerbelli, Simone Scagnoli, Edoardo Cerbelli, Ilaria Grazia Zizzari, Carlo Della Rocca, Giulia D’Amati, Antonella Polimeni, Marianna Nuti, Marco Carlo Merlano, Silvia Mezi, Paolo Marchetti

**Affiliations:** ^1^Department of Clinical and Molecular Oncology, “Sapienza” University of Rome, Rome, Italy; ^2^Department of Radiological, Oncological, and Anatomo-Pathological Science “Sapienza”, University of Rome, Rome, Italy; ^3^Medical Physics Unit, “S. Orsola-Malpighi” Hospital, Bologna, Italy; ^4^Department of Experimental Medicine, University Sapienza, Rome, Italy; ^5^Department of Medico-Surgical Sciences and Biotechnology, Polo Pontino, Sapienza University, Roma, Italy; ^6^Odontostomatological and Maxillo-Facial Science, ‘Sapienza’ University of Rome, Rome, Italy; ^7^Medical Oncology, Candiolo Cancer Institute, FPO-IRCCS, Candiolo (Turin), Italy

**Keywords:** metastatic head and neck cancer, immunotherapy, anti–PD-1, anti–PD-L1, network meta-analysis

## Abstract

**Objective:**

The monoclonal antibodies anti-programmed death protein-1 (anti–PD-1) nivolumab and pembrolizumab are the first immune checkpoint inhibitors (ICIs) approved for treatment of recurrent/metastatic head and neck carcinoma R/M HNSCC in first line and in platinum refractory disease. This network meta-analysis aims to investigate the efficacy of anti–PD-1- *vs* anti–PD-L1-based therapy in R/M HNSCC cancer patients through a systematic review of the literature to provide support for evidence-based treatment decisions. In particular, the effectiveness of ICIs for R/M HNSCC is analyzed according to the different mechanisms of action of the check-points inhibitory drugs in different subgroups of patients.

**Methods:**

We did a systematic literature review and network meta-analysis (NMA) of randomized controlled trials (RCTs) in PubMed, ClinicalTrials.gov, Embase, Medline, the Cochrane Central Register of Controlled Trials, Web of Science. Our search identified a total of five randomized controlled trials: Keynote 040, Keynote 048, Eagle, Condor, Checkmate 141. These trials included 3001 patients. Treatment was sub-categorized into PD-L1–based, PD-1–based, and standard chemotherapy. Treatments were indirectly compared with anti–PD-L1-based therapy.

**Results:**

The network meta-analysis demonstrated no significant differences in OS between different subgroups except for the metastatic patients in which anti–PD-1-based therapy was associated with significantly less risk of death. Furthermore, anti–PD-1-based therapy appeared to be effective in smoker patients and in human papilloma–negative (HPV) patients. Conversely, anti–PD-L1-based therapy seems to be better efficient in female patients, in locally recurrent setting and in HPV positive patients.

**Conclusion:**

This is the first NMA study that aimed to indirectly compare anti–PD-1- and anti–PD-L1-based therapy in HNSCC patients. The results of our NMA could help define a profile of patient responder or resistant to specific classes of immune drugs and can be used to guide/design future studies in the novel scenario of precision immune-oncology.

## Introduction

Head and neck squamous cell carcinomas (HNSCC) represent the sixth most common type of cancer with 830,000 new cases and around 430,000 deaths each year worldwide (1). HNSCC is a spectrum of malignancies arising from the mucosal lining of the upper aerodigestive tract, with different localizations (concerning larynx, hypopharynx, oropharynx, nasopharynx, oral and nasal cavities, and paranasal sinuses) (2, 3). HNSCC is mostly diagnosed at an advanced stage involving loco-regional lymph nodes. Approximately 10% of patients with locally advanced disease already have distant metastases at initial presentation (4). Moreover, despite the aggressive local treatment carried out with radical intent, local and/or distant relapse occurs in more than half of locally advanced HNSCC (5–7).

HNSCC can be classified into human papillomavirus associated (HPV-positive) and HPV-negative sub-types characterized by a different prognostic profile, strongly associated with the oropharyngeal carcinoma and with smoking habit and alcohol consumption, respectively (8–10).

The monoclonal antibodies anti-programmed death protein-1 (anti–PD-1) nivolumab and pembrolizumab are the first immune checkpoint inhibitors (ICIs) approved for treatment of platinum refractory HNSCC recurrent/metastatic (R/M) (11, 12). These immunotherapeutic agents act by enhancing immune system response by blocking suppressive signals through the PD-1/PD-L1 pathway (13, 14).

The results of KEYNOTE-048 trial led to the approval of pembrolizumab in association with cisplatin/5 fluorouracil chemotherapy or as a single agent, in first-line setting in patients whose tumors show a PD-L1 combined positive score (CPS) ≥ 1% (15).

Anti–PD-1 agents have changed the management of HNSCC R/M, based on chemotherapeutic and targeted agents (16–19), becoming the current standard of care. Despite the anti-PD-1 antibodies providing a benefit in terms of tumor progression control and overall survival (OS) compared with chemotherapy (11, 13, 14, 20), overall response still remains limited.

Furthermore, the phase III EAGLE trial (21) and the phase II CONDOR trial (22) investigated the role of durvalumab, alone or in combination with cytotoxic T-lymphocyte-associated antigen 4 (CTLA-4) tremelimumab, versus chemotherapy (23).

Both clinical trials failed to show a statistically significant advantage of durvalumab-based immunotherapy in terms of OS, even though the immunotherapy strategy showed higher response rate and survival rates at 12 to 24 months, highlighting the clinical activity of durvalumab.

Data from clinical trials that investigate ICIs in HNSCC showed that only a relatively small subset of patients really benefit from treatment, underlining the crucial role of patients’ selection before starting immunotherapy (24).

Therefore, a deeper understanding of immune resistance mechanisms, probably dependent to the specific check point inhibitor mechanism of action, is urgently needed.

The response to immunotherapy could be affected by the features of tumor microenvironment (TME) (25–28) that is potentially different between primary tumors, primary tumor, and metastatic sites and finally between different sites of metastasis (29, 30).

The evaluation of clinical characteristic of patients should be considered. Indeed, several factors, such as age and gender (31–33), have shown an important role in conditioning the response to immunotherapy resulting in novel predictive biomarkers.

This network meta-analysis aims to investigate the efficacy of anti–PD-1- *vs* anti–PD-L1-based therapy in HNSCC cancer patients through a systematic literature review (including data from the most recent randomized controlled trials) to provide support for evidence-based treatment decisions.

In particular, the effectiveness of ICIs for advanced or metastatic HNSCC is analyzed according to different subgroups of patients (in relation to baseline characteristics) and to the different mechanisms of action of the check-points inhibitory drugs.

To the best of our knowledge, this is the first study indirectly comparing the effect of anti–PD-1 and anti–PD-L1 therapy in HNSCC patients.

## Materials and Methods

We performed a systematic literature review and network meta-analysis (NMA) of randomized controlled trials (RCTs) in PubMed, ClinicalTrials.gov, Embase, Medline, the Cochrane Central Register of Controlled Trials, and Web of Science. Conference abstracts from the American Society of Clinical Oncology (ASCO) and ESMO were searched independently. Only English language publications were included. The search covered the literature up to July 2017.

Search terms included the following: randomized clinical trials, locally advanced and metastatic head and neck cancer, immunotherapy, anti–PD-1, and anti–PD-L1. Search results were restricted to phase II and phase III RCTs.

Bibliographies of review articles and editorials were manually searched. The literature review process followed Preferred Reporting Items for Systematic Reviews and Meta-Analyses (PRISMA) guidelines (34). Two authors independently evaluated data from eligible studies, which were then checked by a third author.

We performed an NMA for OS data using a random-effects model with a frequentist approach (35, 36) to account for this potential heterogeneity (different study designs, populations, treatment arms, etc.). Treatments were ranked by calculating P scores using the netrank function of the netmeta R-package (37, 38). P scores measure the extent of certainty that a treatment is better than another treatment, averaged over all competing treatments, while taking the precision into account (38). In our study, we used a p<0.05 threshold to judge the statistical significance of our findings, which means that the results are statistically significant if the confidence intervals do not include the value of 1 (for HR and relative risk). We also used a p<0.10 threshold as trend because of the reduced number of patients in the various investigated subgroups. The forest plot, with the HR being<1, is indicative of inferior efficacy of all other treatments compared with anti–PD-L1-based therapy.

The odds ratio as a simple percent increase or decrease of an event happening, as this value depends on the base-rate, was evaluated according to the following formula:

$$Ptreatment=\frac{OR\times Pcontrol}{1+OR\times Pcontrol-Pcontrol}$$

## Results

**Figure 1:**
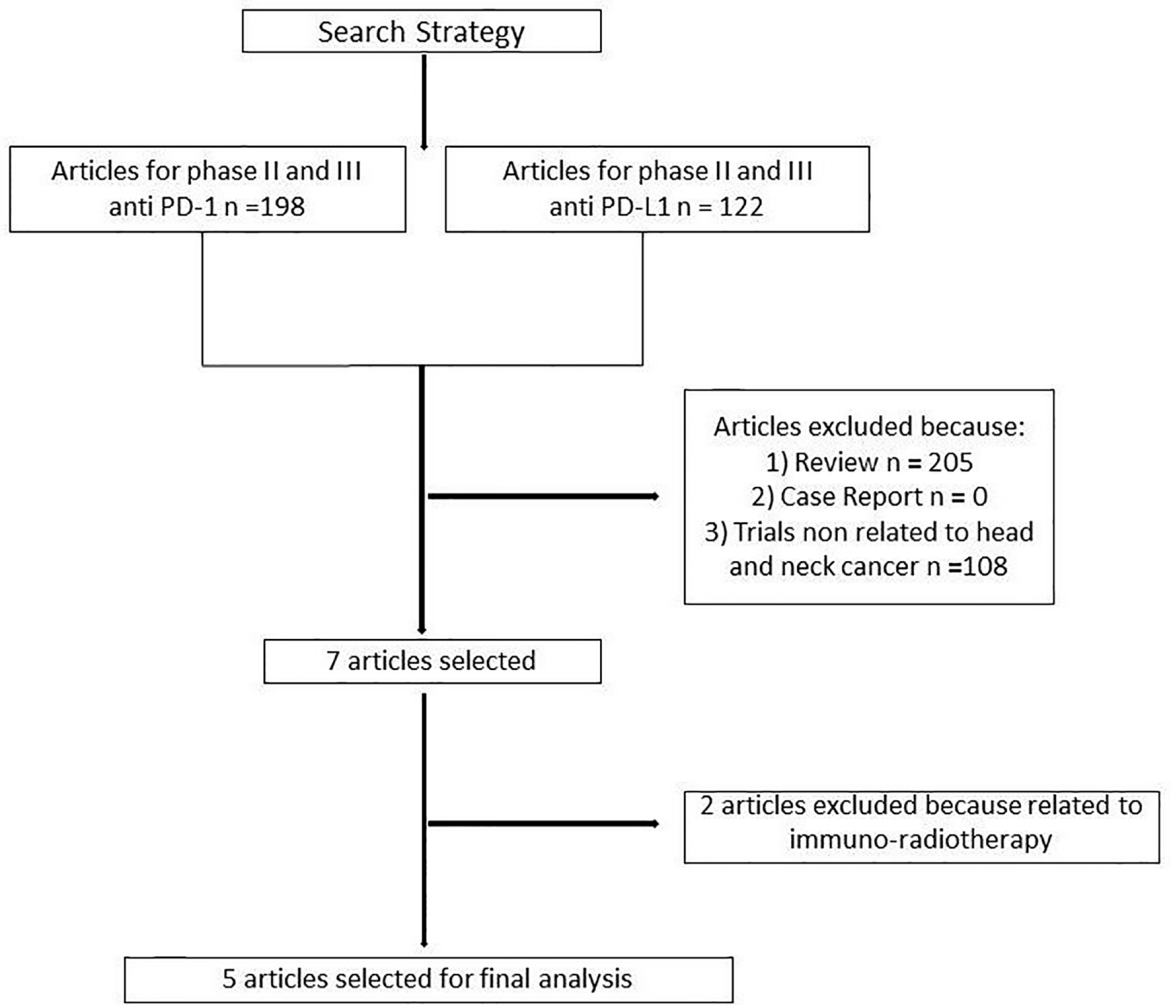
Diagram of selection process for trials included in meta-analysis.

**Figure 2:**
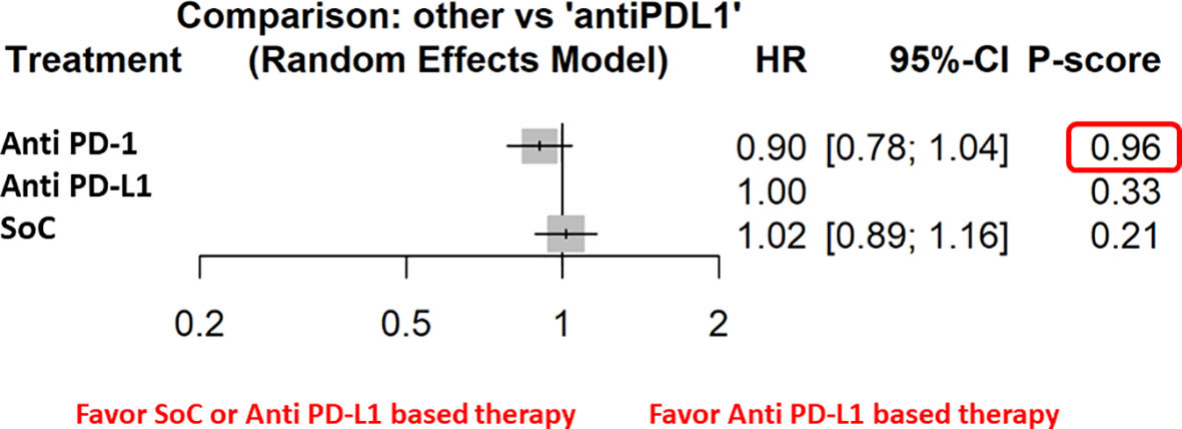
The NMA results of the indirect efficacy comparison of anti–PD-1 and SoC with anti–PD-L1 in the whole population.

One hundred and ninety-eight articles were selected for phase II and III clinical trials anti–PD-1 therapy and 122 for anti–PD-L1 therapy. Three hundred and thirteen articles were analyzed. Three hundred and six articles were excluded because non randomized trials, review, or not related to head and neck cancer. Two further trials were excluded because related to immuno-radiotherapy (**Figure 1**). Our search identified a total of five randomized controlled trials: Keynote 040, Keynote 048, Eagle, Condor, Checkmate 141. These trials included 3001 patients (**Table 1S**). Treatment was sub-categorized into PD-L1–based and PD-1–based and the standard chemotherapy (**Figure 2**). Treatments were indirectly compared with anti–PD-L1-based therapy. The patient’s characteristics, from the identified RCT, are summarized in **Table 1**.

**Table 1:** Patients’ characteristics of the identified RCTs.

	Subgroups	N tot. (%)	Anti–PD-1	Anti–PD-L1
**All patients**		3001 (100%)	2016 (100%)	985 (100%)
**Sex**	Female	426 (14%)	266 (13%)	160 (16%)
	Male	2214 (74%)	1389 (69%)	825 (84%)
	Not reported	361 (12%)	361 (18%)	0 (0%)
**Age**	<65 years	2011 (67%)	1326 (66%)	685 (70%)
	≥65 years	990 (33%)	690 (34%)	300 (30%)
**Disease**	Metastatic	1246 (42%)	807 (40%)	439 (45%)
	Recurrent only	774 (26%)	340 (17%)	434 (44%)
	Not reported	981 (33%)	869 (43%)	112 (11%)
**ECOG**	0	947 (32%)	663 (33%)	284 (29%)
	≥1	2051 (68%)	1350 (67%)	701 (71%)
	Not reported	3 (0%)	3 (0%)	0 (0%)
**HPV-status**	Positive	492 (16%)	370 (18%)	122 (12%)
	Negative	1523 (51%)	1285 (64%)	238 (24%)
	Not reported	986 (33%)	361 (18%)	625 (64%)
**Smoking habit**	Never	586 (20%)	378 (19%)	208 (21%)
	Former	1598 (53%)	1019 (50%)	579 (59%)
	Current	452 (15%)	254 (13%)	198 (20%)
	Not reported	365 (12%)	365 (18%)	0 (0%)

The network meta-analysis of OS demonstrated no significant differences between different subgroups except for the metastatic patients in which anti–PD-1-based therapy was associated with significantly less risk of death. In addition, our results showed a benefit in terms of OS in the male population and in patients with current smoking habit.

**Figure 3:**
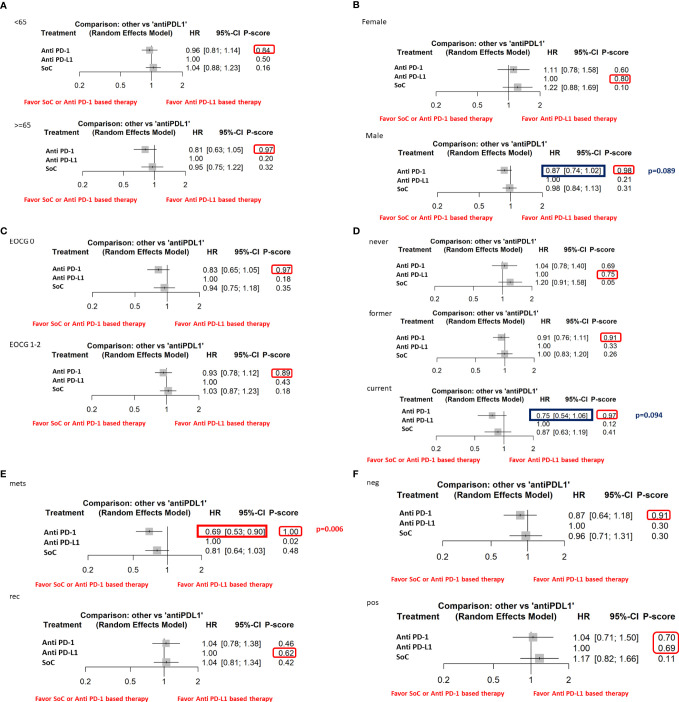
The NMA results of the indirect efficacy comparison of anti–PD-1-based therapy and SoC with anti–PD-L1-based therapy in all the available subgroups: **(A)** age, **(B)** sex, **(C)** ECOG PS, **(D)** smoke habit, **(E)** type of disease, **(F)** HPV status.

Furthermore, the indirect analysis revealed that the anti–PD-1-based therapy had the highest probability of being the best treatment in the whole population (P score = 0.96), in male patients (P=0.98), in metastatic patients (P=1), in negative HPV cancer patients (P=0.91), in patients with former (P=0.91), and current (P=0.97) smoking habit. The analysis evidenced that OS was irrespective of ECOG PS [both ECOG performance status = 0 (P=0.97) and 1-2 (P=0.89)] and patient’s age [patients with age higher (P=0.97) or lower (P=0.84) than 65 years] (**Figure 3**).

Moreover, the indirect analysis revealed that anti–PD-L1-based therapy had the highest probability of being the best treatment in patients never smoking (P=0.75), with local recurrence disease (P=0.62) or in the female subgroup. In the subgroups of patients with positive HPV status, anti–PD-1- (P=0.70) and anti–PD-L1-based therapy (P=0.69) had a similar probability of being the best treatment highlighting a higher efficacy than the chemotherapy treatment.

## Discussion

The therapeutic arsenal of HNSCC is rapidly evolving because of the introduction of new immunotherapeutic agents, which have been shown to improve treatment outcomes and OS in recurrent and metastatic disease as single agents, as well as in combination with chemotherapy.

However, only a small percentage, about 20% to 30%, of HNSCC patients presented a long-term benefit from immunotherapy.

In the context of several available treatments, the selection of patients prone to respond to chemotherapy or immunotherapy could be crucial to define the combination or a sequential approach.

Unfortunately, only PD-L1 expression is currently used in our clinical practice, so lack of biomarkers led us to better consider the clinical features to guide the choice of strategy (39).

This NMA was conducted to provide a comprehensive comparison of the efficacy of anti–PD-1-based therapy or anti–PD-L1-based therapy for advanced and/or metastatic HNSCC patients and in several subgroups compared with chemotherapy treatment. However, several treatments had P scores >50%, and the confidence intervals demonstrate no significant differences between anti–PD-1-based anti–PD-L1-based therapy.

It should be emphasized that when an NMA analysis is undersized (with a large 95% CI), it cannot be defined as “similar efficacy” between two treatments. Although we have not demonstrated statistically significant differences in the efficacy of anti–PD-1 therapy versus anti–PD-L1 therapy in the whole population, this does not rule out the possibility of an advantage when analyzing specific or larger subgroups.

In particular anti–PD-L1-based therapy seems to be more efficient in female patients, in recurrent setting, and in HPV-positive patients.

The gender effect on the response to immunotherapy was widely investigated in several studies, such as the different susceptibility of autoimmune disease according to the reproductive status.

Despite the controversial results of durvalumab alone or in combination with tremelimumab in Eagle and Condor studies, two recent metanalysis demonstrated the benefit of anti–PD-L1-based therapy, in terms of survival and safety, in recurrent disease, suggesting a specific role in this setting that could be immunologically different from the metastatic ones (40, 41). Furthermore, durvalumab demonstrated higher efficacy, in terms of response rate and survival, in HPV-positive patients (42), highlighting the putative role of HPV infection in the modulation of immune response creating a more “ready to act” microenvironment (43, 44). Conversely, anti–PD-1-based therapy is more promising in terms of therapeutic efficacy in male and smoker patients. We have already demonstrated the sexist behavior of anti–PD-1 treatment in favor of male patients (45), and several studies highlighted the strong association between response to immunotherapy and smoking status, regardless of the type of cancer (46–49). Indeed, the effect of smoke on mutation of DNA could lead to an increased tumor mutational burden (TMB) with an impact on immunogenicity especially if non-synonymous mutations are involved (50–52). In addition, our meta-analysis suggests the higher benefit of anti–PD-1-based therapy in metastatic patients. We suggest that the subgroup of metastatic patients’ anti–PD-1-based therapy was associated with lower risk of death. Based on the information (number of patients in the subgroups and overall number of death) data, our results suggest a risk reduction in the metastatic patients of 3.1% using the anti–PD-1-based therapy, whereas the reduction was not significant between SoC and anti–PD-L1. The median OS was 8.7 and 7 months for patients PD-L1–positive and PD-L1–negative, respectively. Unfortunately, the median OS was not reported for all the subgroups except for HPV-positive and HPV-negative ones, resulting in 6.75 and 6.65 months, respectively, suggesting a higher benefit of anti–PD-1-based therapy in HPV-negative patients.

These results, in contrast with the effect of anti–PD-L1, could be explain by the different monoclonal antibodies targets. In particular, the PD-1 therapy effect is mediated by the binding with T lymphocytes (53–55) resulting in a systemic effect, whereas the activity of anti–PD-L1 therapy is directed against the receptor expressed on tumor cells (56, 57) determining a localized effect.

It is well known that metastasis is characterized by a colder microenvironment and that different sites of metastasis present heterogeneous expression of PD-L1 (58, 59) and TILs (29, 60–63). The heterogeneity of PD-L1 expression and the peculiar immunological behavior of each metastatic site affect the response to immunotherapy (64, 65), identifying some site as “immunologically sanctuary” organs.

This speculation may lead to explain the reason why anti–PD-L1-based therapy could be more effective in advanced or recurrent disease, in which PD-L1 expression is less heterogeneous, whereas anti–PD-1, acting on T lymphocytes, is more effective in metastatic disease, independent from the specific site of metastasis and local microenvironment (53–55).

A limitation of this analysis is that only five RCTs are included in this study (because of the recent introduction of immunotherapy in the head and neck cancer). A further limitation is that the five included RCTs are of open-label design and were supported by pharmaceutical industry funding, and finally, immunotherapy treatment is included regardless of (I) the line of therapy, (II) the level of PD-L1/PD-1 expression, and (III) the conventional therapies received in the different clinical trial groups.

Nevertheless, the results of our NMA could help define a profile of patient responder (66, 67) or resistant (68) and can be used to guide/design of future studies in the novel scenario of precision immune-oncology (69).

Limitations of meta-analyses using pooled/aggregate data have been discussed previously (70). As the confidence intervals in our analysis and other published NMAs (70, 71) are relatively wide, results need to be treated with caution.

## Conclusions

**Figure 4:**
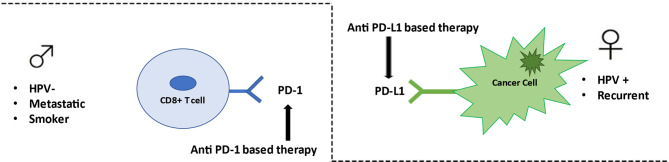
Anti–PD-1/PD-L1 therapy in HN cancer. Anti–PD-1-based therapy appears to be effective in metastatic patients, smoker patients, and HPV-negative patients. Conversely anti–PD-L1-based therapy seems to be better efficient in female patients, in recurrent setting, and in HPV positive patients.

This is the first NMA study aiming at indirectly comparing anti–PD-1- and anti–PD-L1-based therapy in HNSCC patients. Our analysis suggests that there are no statistically significant differences in the efficacy among anti–PD-1- and anti–PD-L1-based therapy, with the exception of subgroup of metastatic patients, in which anti–PD-1-based therapy was associated with significantly lower risk of death. Although not reaching statistical significance, our study suggests a different effect of anti–PD1-based and anti–PD-L1-based therapy in female, with respect to male or HPV-positive or -negative, and in recurrent or metastatic setting (**Figure 4**). Our findings may bolster information from pairwise comparisons to shape HNSCC clinical decision making and to assist planning of future RCTs. A comprehensive evaluation based on immune differences between genders, extent of disease, HPV status, smoking habits, together with new predictive molecular biomarkers may determine selecting the most appropriate type of immunotherapy treatment in the future, allowing the personalization of treatments and finally applying the principle of precision medicine.

## Data Availability Statement

The original contributions presented in the study are included in the article/**Supplementary Material**. Further inquiries can be directed to the corresponding author.

## Author Contributions

Conception and design: AB, PM, and SM. Wrote the manuscript: AB and AC. Acquired data: LS and AC. Analyzed the data: LS. Discussed the results and implications of findings: AB, PM, MM, SM, and AC. Interpretation of data: AB, SM, and AC. Drafting of the manuscript: AB and AC. All authors contributed to the article and approved the submitted version.

## Conflict of Interest

PM has/had a consultant/advisory role for BMS, RocheGenentech, MSD, Novartis, Amgen, Merck Serono, Pierre Fabre, and Incyte.

The remaining authors declare that the research was conducted in the absence of any commercial or financial relationships that could be construed as a potential conflict of interest.

## Publisher’s Note

All claims expressed in this article are solely those of the authors and do not necessarily represent those of their affiliated organizations, or those of the publisher, the editors and the reviewers. Any product that may be evaluated in this article, or claim that may be made by its manufacturer, is not guaranteed or endorsed by the publisher.

## Supplementary Material

Supplementary Table 1 PRISMA checklist: characteristics of selected studies for final analysis.
