# Supplementary figures and images for: Anti–PD-1 and Anti–PD-L1 in Head and Neck Cancer: A Network Meta-Analysis
Source: Front Immunol. 2021 Aug 9;12:705096. doi: 10.3389/fimmu.2021.705096 (PMC8380817; doi:10.3389/fimmu.2021.705096)

**
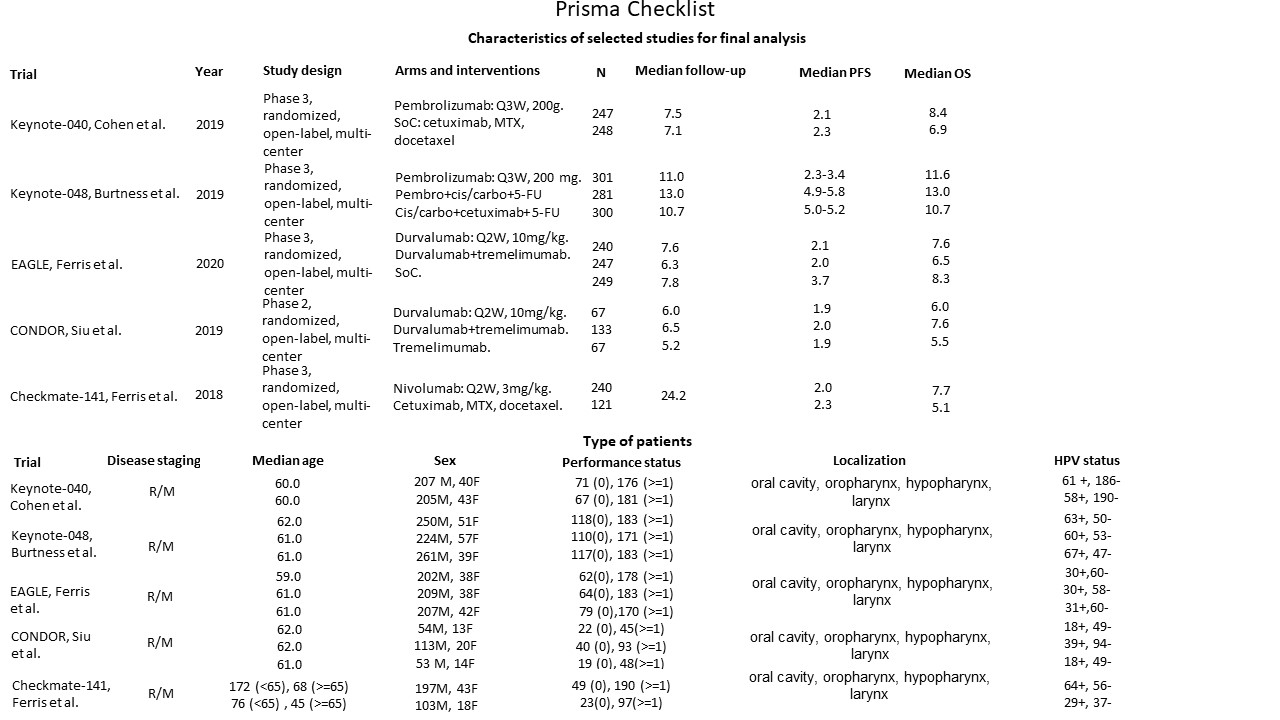
Table 1s**: PRISMA checklist: characteristics of selected studies for final analysis

Supplement: Supplementary Table 1 — PRISMA checklist: characteristics of selected studies for final analysis. [file DataSheet_1.docx]
